# Aging increases the expression of lung CINCs and MCP-1 in senile patients with pneumonia

**DOI:** 10.18632/oncotarget.21285

**Published:** 2017-09-27

**Authors:** Wei Li, Cheng Ding, Shaojun Yin

**Affiliations:** ^1^ Department of Geriatrics, Shanghai Tenth People's Hospital Affiliated to Tongji University, Tongji University School of Medicine, Shanghai, 200072, China; ^2^ General Practitioner, Dachang Community Health Service Center, Dachang Town, Baoshan District, Shanghai, 200442, China; ^3^ Department of Respiratory Medicine, Shanghai No.6 People's Hospital, Shanghai, 201306, China

**Keywords:** pneumonia, senile, MCP-1, CINC-1, CINC-2α

## Abstract

**Objective:**

To explore the relationship between aging and the expression of monocyte chemoattractant protein (MCP) and cytokine-induced neutrophil chemoattractant (CINCs) in patients with pneumonia.

**Results:**

Bacteria counts in senile group were significantly higher than non-senile group, and while white blood cell and neutrophil counts in senile group were observably lower than non-senile group. The concentration of MCP-1 was significantly higher in senile group compared with the non-senile group, and the expression of CINC-1 and CINC-2α was also higher in senile group. In all patients with different pathogens, expression of all the factors was significantly higher in senile group compared with the non-senile group. What’s more, expression of MCP-1, CINC-1 and CINC-2α showed significant difference in some patients with different pathogens. CINC-2β and CINC-3 expression was not detected in both groups.

**Materials and methods:**

The present study included 800 patients with pneumonia who were hospitalized to the Department of Respiratory Medicine in Tongji Hospital during the period from December of 2014 to June of 2016. All patients were divided into two groups: senile pneumonia and non-senile pneumonia group. Bacteria, white blood cell and neutrophil counts were determined by automatic blood cell analyzer. The expression of MCP-1, CINC-1, CINC-2α, CINC-2β and CINC-3 was determined by ELISA assay.

**Conclusions:**

Aging can increase the expression of MCP-1,CINC-1 and CINC-2α in patients with pneumonia, which may lead to increased risk of pneumonia in the elderly.

## INTRODUCTION

Pneumonia is the leading infectious cause of death and the fourth overall cause of mortality in the elderly [1]. It is considered that risk factors of underlying comorbid diseases, impaired mucociliary clearance and waning immunity have contributed to the increased incidence of pneumonia in the elderly individuals [2]. In recent years, with increasing socio-demographic aging, the number of aging infected patients has quickly increased, becoming a global problem. Since lungs are an open gate to invasion by bacteria, pneumonia occurs with particularly high frequency and ranks first among the causes of death for aged people [3].

The family of C-X-C chemokines is a kind of potent neutrophil chemoattractants that have been implicated in neutrophil influx to acute inflammatory sites [4]. Previous studies have shown that both cytokine-induced neutrophil chemoattractant (CINC) and CINC-3/macrophage inflammatory protein (MIP)-2 are increased in lung in response to LPS, which can induce lung inflammation [5, 6]. It has been proved that rat CINC-1, CINC-2 and CINC-3/MIP-2, members of the CXC chemokine family, are potent chemotactic factors for neutrophils [7]. CINCs are particular members of the CXC family that are active as neutrophil chemoattractants *in vitro* and *in vivo*. It is known to be induced in the rat in response to tumor necrosis factor (TNF), IL-1, and lipopolysaccharide (LPS) [8, 9]. Monocyte chemoattractant protein-1 (MCP-1) proteins are small (8-10 kDa), structurally related, inducible, secreted, and proinflammatory chemokines of the CC subfamily [10]. It is the major lymphocyte chemoattractant secreted by mitogen-stimulated peripheral blood mononuclear cells and acts as a potent T-lymphocyte and monocyte chemoattractant. Recently, a study showed that MCP-1 could be a potential Predictor of the Severity of Community-Acquired Pneumonia [11]. Animal studies also demonstrated that MCP-1 and CINCs are related with pneumonia [12]. However, clinical evidences for relationship between expression of CINC and MCP-1 and pneumonia patients with different ages are still lacking.

In this study, we firstly determined the relationship between the expression of MCP-1 and CINCs and aging in pneumonia patients. This study may provide more clinical evidence for further understanding relationship of aging and MCP-1 and CINCs in pneumonia patients.

## RESULTS

### Etiology analysis on pneumonia

In 300 cases of senile pneumonia patients, 211 strains of pathogen were separated. Among the pathogens, there were 130 strains of streptococcus pneumoniae, 9 strains of haemophilus influenza, 22 strains of aspiration pneumonia, 17 strains of legionella pneumophila, 1 strain of staphylococcus aureus, 7 strains of gram-negative bacilli and 6 strains of atypical agents. Among the streptococcus pneumoniae, there were 18 strains of pneumococcal bacteremia and also 8 strains of pseudomonas aeruginosa included in gram-negative bacilli. In 500 cases of non-senile pneumonia patients, 325 strains of pathogen were separated. There were 187 strains of streptococcus pneumoniae, 19 strains of haemophilus influenza, 41 strains of aspiration pneumonia, 30 strains of legionella pneumophila, 2 strains of staphylococcus aureus, 10 strains of gram-negative bacilli and 13 strains of atypical agents. Among the streptococcus pneumoniae, there were 40 strains of pneumococcal bacteremia and also 14 strains of pseudomonas aeruginosa included in gram-negative bacilli. The difference of pathogen in senile pneumonia and non-senile pneumonia group had no significant difference (Table 1).

**Table 1:** Etiology of pneumonia in senile and non-senile group

Variable	Senile (*n* = 300) No.(%)	Non-senile (*n* = 500) No.(%)	*p*
Streptococcus pneumoniae	130 (43.3)	187 (37.4)	0.21
Pneumococcal bacteremia	18 (6)	40 (21.3)	0.15
Haemophilus influenzae	9 (3)	19 (3.8)	0.36
Aspiration pneumonia	22 (7.3)	41 (8.2)	0.61
Legionella pneumophila	17 (5.7)	30 (6)	0.12
Staphylococcus aureus	1 (0.3)	2 (0.4)	0.53
Gram-negative bacilli	8 (2.7)	14 (2.8)	0.39
Pseudomonas aeruginosa	7 (2.3)	10 (2.0)	0.43
Atypical agents	6(2)	13 (2.6)	0.27
Other	5 (1.7)	11 (2.2)	0.36
No pathogen identified	95 (31.7)	175 (35)	0.12

### Bacteria, white blood cell and neutrophil counts in BAL fluid

Bacteria cause approximately half of all chronic obstructive pulmonary disease exacerbations [13]. Sequential exposure to bacteria enhances pulmonary inflammation and infectivity [14]. Therefore, we determined the number of bacteria, white blood cell and neutrophil in BAL supernatant. As shown, the bacteria counts in lung tissue from the senile group were significantly higher than the non-senile group (*p < 0.05*). The total number of white blood cells and neutrophil in the blood of the senile group was significantly lower than in the non-senile group (*p < 0.05*) (Table 2).

**Table 2:** White blood cells counts in BAL fluid of senile and non-senile group

Counts	Senile (×10^4^ CFU/ml mean ± SD)	Non-senile (× 10^4^ CFU/ml mean ± SD )	*P*
Bacteria	5.23 ± 1.12	9.15 ± 2.33	0.031
White blood cell	4.62 ± 1.31	10.75 ± 2.56	0.018
Neutrophil	4.81 ± 1.05	12.04 ± 4.29	0.013

### Expression of MCP-1and CINCs

**Figure 1:**
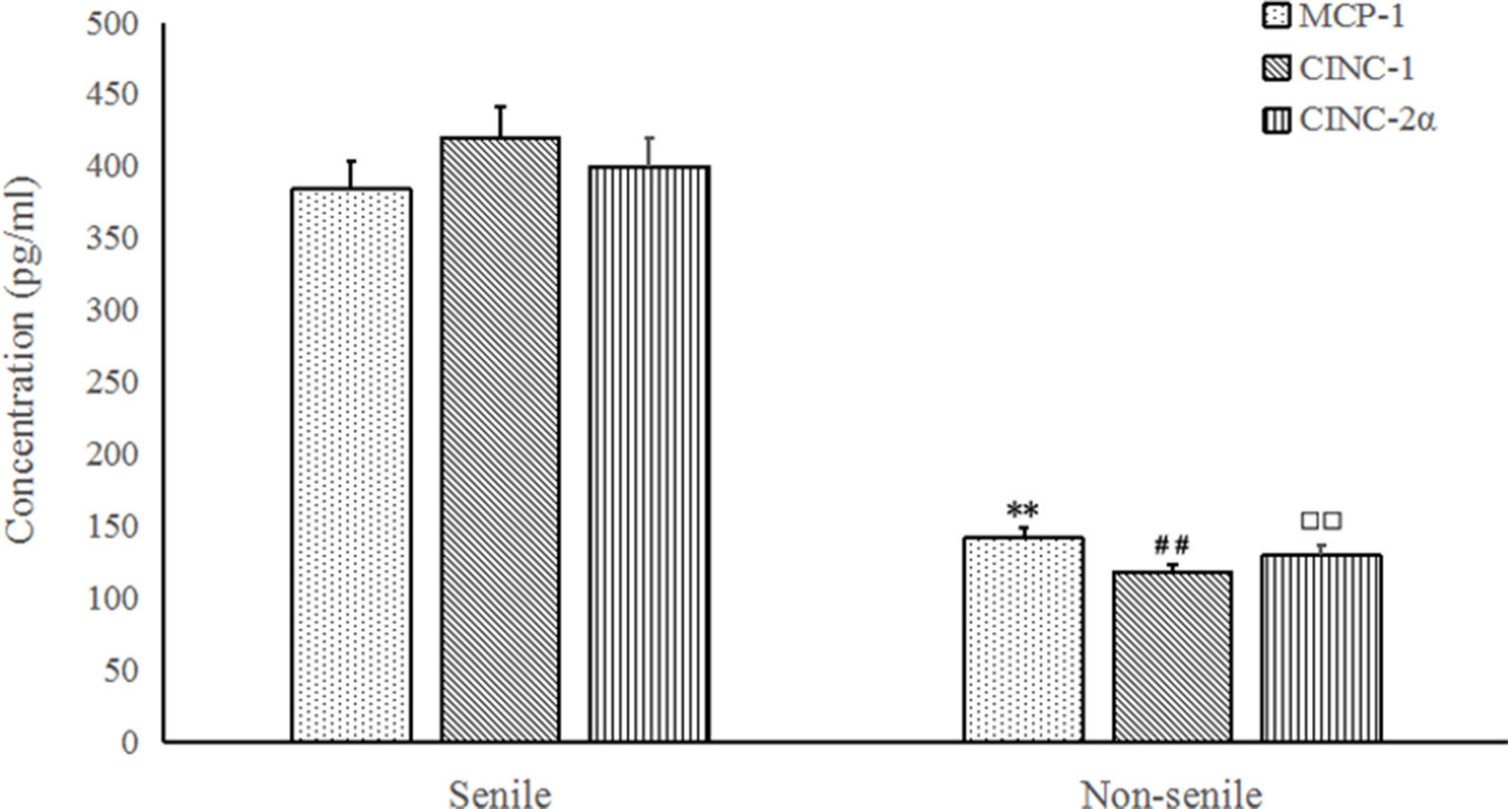
MCP-1 (A), CINC-1 (B) and CINC-2α (C) protein concentrations in BALF from senile and non-senile pneumonia patients *P < 0.05, compared with the concentration of MCP-1 in senile group; ^#^*P* < 0.05, compared with the concentration of CINC-1 in senile group; ^□^*P* < 0.05, compared with the concentration of CINC-2α in senile group.

MCP-1 has been implicated in the recruitment of different leukocyte subsets to sites of infection and inflammation [15]. CINC-1, CINC-2α, CINC-2β, CINC-3 have a comparable neutrophil chemotactic activity and the ability to induce cathepsin G release from rat neutrophils *in vitro*. To determine the relationship of expression of MCP-1 and CINCs with patients in saline and non-saline groups, serum levels of MCP-1 and CINCs were measured. Results showed the concentration of MCP-1 was significantly up-regulated in senile group compared with non-senile group (*p < 0.05*) (Figure 1). The expression of CINC-1 and CINC-2α was found in both group, and the value in senile group was significantly higher than non-senile group (*p < 0.05*). The expression values of serum CINC-2β and CINC-3were not detected in both groups.

### Influence of pathogens on expression of MCP-1 and CINCs in different groups

To explore whether different pathogens in pneumonia patients resulted in the different expression of MCP-1, CINC-1 and CINC-2α in senile and non-senile patients, we determined the expression of MCP-1, CINC-1 and CINC-2α in patients with different pathogens. As shown in Table 3, in all patients with different pathogens, expression of all the factors were significantly higher in senile group compared with the non-senile group (*p < 0.05*). What’s more, expression of MCP-1, CINC-1 and CINC-2α showed significant difference in some patients with different pathogens. Expression of MCP-1 was significantly lower in patients with aspiration pneumonia in senile patients and was significantly lower in patients with aspiration pneumonia, legionella pneumophila and pseudomonas aeruginosa in non-senile patients (*p < 0.05*). Expression of CINC-1 was significantly lower in patients with streptococcus pneumoniae and legionella pneumophila in senile patients (*p < 0.05*), but was significantly higher in patients with streptococcus pneumonia and pneumococcal bacteremia in non-senile patients (*p < 0.05*). At last, expression of CINC-2α was significantly lower in patients with legionella pneumophila in senile patients and was significantly higher in patients with pneumococcal bacteremia in non-senile patients.

**Table 3:** Expression of MCP-1 in different aging and pathogen

	Senile (pg/ml mean ± SD)	Non-senile (pg/ml mean ± SD)	*P*
MCP-1			
Streptococcus pneumoniae	352.7 ± 21.4	135.3 ± 18.7	0.028
Pneumococcal bacteremia	361.5 ± 23.5	137.1 ± 20.0	0.014
Aspiration pneumonia	328.6 ± 15.6	115.4 ± 11.7	0.010
Legionella pneumophila	371.4 ± 22.4	98.2 ± 8.3	0.008
Pseudomonas aeruginosa	381.6 ± 11.2	94.1 ± 5.1	0.012
CINC-1			
Streptococcus pneumoniae	421.6 ± 15.8	156.2 ± 9.5	0.017
Pneumococcal bacteremia	452.7 ± 13.8	172.0 ± 10.2	0.021
Aspiration pneumonia	430.6 ± 11.7	118.9 ± 8.2	0.007
Legionella pneumophila	418.5 ± 16.3	116.2 ± 7.4	0.015
Pseudomonas aeruginosa	460.5 ± 18.3	130.6 ± 9.1	0.017
CINC-2α			
Streptococcus pneumoniae	486.3 ± 23.1	106.8 ± 9.5	0.026
Pneumococcal bacteremia	461.9 ± 19.4	142.7 ± 10.5	0.031
Aspiration pneumonia	438.2 ± 17.5	128.3 ± 10.7	0.028
Legionella pneumophila	395.8 ± 15.3	119.3 ± 13.5	0.019
Pseudomonas aeruginosa	405.7 ± 20.1	130.4 ± 12.5	0.021

## DISCUSSION

Pulmonary inflammation, particularly in susceptible individuals, may be an important mechanism by which ambient particles may adversely affect human health. Blood neutrophils (PMN) trafficking during inflammation is a complex process which involves endothelial and PMN adhesion molecules and involvement of several types of chemotactic factors which may include complement activation products, and especially CXC chemokines [16, 17]. Recently, relationship between chemokines and pneumonia has attracted scholars’ attention. Some new studies have demonstrated that CINCs and MCP-1 play important roles in development of pneumonia [11, 12]. However, to our best knowledge, no clinical evidences have been given for relationship of aging and expression of CINCs and MCP-1. In the present study, we conducted a clinical investigation to demonstrate the possible link between the chemokines.

Following with the determination of pathogens and analysis of BAL fluid, expression of MCP-1 and CINCs in saline and non-saline groups were measured. Results showed that the concentration of MCP-1 was significantly up-regulated in senile group compared with non-senile group, and the expression of CINC-1 and CINC-2α was significantly higher than non-senile group. The expression values of CINC-2β and CINC-3 were not detected in both groups. An animal study was reported recently, Wen et al demonstrated that aging could reduce the expression of lung CINC and MCP-1 in a *P. aeruginosa* rat model of infection [12]. However, in the present study we found in senile patients CINCs and MCP-1 were up-regulated. The difference might be because of the infection period. In Wen’s study, both expression of MCP-1 and CINCs was lower within 24 h in aged rats but was higher at the time point of 24 h, which was in consistent with our study to a certain extent.

Lots of studies have reported that CINC and MCP-1 was up-regulated in animal models of lung infection or lung injury. Kim et al. showed that valproic acid could reduce expression of CINC in intestinal ischemia reperfusion rats [18]. Jr et al demonstrated that MCP-1 was significantly increased in inflammatory disorders of the lung in animal models [19]. Recently, Yong et al firstly showed that serum MCP-1 was also up-regulated in pneumonia patients [11]. However, whether aging can affect expression of MCP-1 and CINC in pneumonia patients have not been studied yet.

To further study influence of different pathogens on expression of the factors, we then determined levels of MCP-1, CINC-1 and CINC-2α in patients with different pathogens. Results showed that in all patients with different pathogens, expression of all the factors were significantly higher in senile group compared with the non-senile group. What’s more, expression of MCP-1, CINC-1 and CINC-2α showed significant difference in some patients with different pathogens. However, deeper understanding of the difference needs further investigation.

In conclusion, the expression values of MCP-1, CINC-1 and CINC-2α in senile pneumonia patients were significantly different with the non-senile pneumonia patients, which can influence the migration of leucocytes and neutrophils.

## MATERIALS AND METHODS

### Patients

Eight hundred patients with pneumonia were selected among all 1139 pneumonia patients in this study who were hospitalized to the Tenth People's Hospital of the elderly, respiratory medicine, emergency department observation room, intensive care unit, and the Sixth People's Hospital of Respiratory Medicine. During the period from December of 2014 to June of 2016. The diagnosis of pneumonia was established on the bases of history, symptoms, physical examination and chest X-ray manifestations. Patients who were within the following criteria were excluded: patients with other severe infections, severe heart diseases or hepatorenal diseases, patients with cancer, pregnant patients, patients who had taken antibiotic therapy 1 month before sutdy, patients younger than 18 and patients who didn’t sign the informed consent. The etiology of pneumonia was determined by one of the following criteria: (1) blood cultures or pleural fluid yielding a bacterial or fungal pathogen in the absence of an apparent extrapulmonary focus; (2) seroconversion (fourfold increase in IgG titers for *C. pneumonia*, *M. pneumoniae*, *L. pneumophila*, and respiratory viruses such as influenza viruses A and B, parainfluenza viruses 1 to 3, and adenovirus); (3) positive urinary antigen for *L. pneumophila* type 1; (4) bacterial growth of ≥ 103 colony-forming units (CFU)/ml of a pathogenic microorganism from a PSB specimen or TNA; growth of ≥ 104 CFU/ml of a pathogenic microorganism from PBAL; or ≥ 105 CFU/ml in BAL. Isolation of fungi from respiratory samples was considered diagnostic only in the presence of positive concomitant blood culture yielding the same organism.

The patients with pneumonia were divided into 2 groups in terms of age. Senile patients group of 300 cases (Group A, 180 males and 120 females, mean age 68 ± 7.13 years) with the age of at least 65 years old, and non-senile patients group of 500 cases (Group B, 270 males and 230 females, mean age 41 ± 11.34 years) with the age ranging from 18 to 65 years old. Informed consents were obtained from all the patients. The study was approved by Ethic Committee of Shanghai Tenth People’s Hospital Affiliated to Tongji University.

### Bronchoalveolar lavage (BAL)

For some patients with refractory pneumonia and severe pneumonia, BAL was used in treatment [20] and bacteria, white blood cell and neutrophil counts in BAL fluid were measured using the CELL DYN3700 automatic blood cell analyzer [21]. For BAL collection, briefly, five separate 30-mL aliquots of 0.89% sterile saline were instilled into the right middle lobe or lingula of left lung. Immediately on collection BALF was centrifuged at 14,000 g for 20 minutes at 4°C and cell-free supernatants aliquoted and stored at –80°C until further assayed.

### Enzyme-linked immunosorbent assay (ELISA)

Serum samples were collected at the beginning of the study when patients were hospitalized. The serum levels of CINC-1, CINC-2α, CINC-2β, CINC-3 and MCP-1 were determined by enzyme-linked immunosorbent assay (ELISA) kits (Nanjing Jiancheng Bioengineering Institute, Nanjing, China) according to manufacturer’s protocol.

### Statistical analysis

All data are expressed as mean ± SD. Differences between groups were analyzed by Mann-Whitney *U* test. Values of *p < 0.05* were considered to represent a statistically significant difference.
